# Skuas as sentinels of high pathogenicity avian influenza H5N1 on the Antarctic Peninsula in the 2024/2025 austral summer

**DOI:** 10.1099/mgen.0.001724

**Published:** 2026-08-14

**Authors:** Michelle Wille, Will Abbott, Darrel Day, Yi-Mo Deng, Xiaomin Dong, Ted Gibson, Ryan Hope-Inglis, Michelle McCulley, Ida Olsson, Arvind Varsani, Teri Visentin, Matthew Walters, Meagan Dewar

**Affiliations:** 1 WHO Collaborating Centre for Reference and Research, at the Peter Doherty Institute for Infection and Immunity, Melbourne, Victoria, Australia; 2 Department of Microbiology and Immunology, University of Melbourne, at the Peter Doherty Institute for Infection and Immunity, Melbourne, Victoria, Australia; 3 Intrepid Travel, Sydney, Australia; 4 Consulting Antarctica, Sydney, Australia; 5 Division of Health, University of Waikato, Private Bag 3105, Hamilton 3240, New Zealand; 6 Biodesign Center for Fundamental and Applied Microbiomics, Center for Evolution and Medicine and School of Life Sciences, Arizona State University, Tempe, USA; 7 School of Life and Environmental Sciences, Deakin University, Geelong, Australia; 8 School of Biological Sciences, University of Canterbury, Christchurch, New Zealand; 9 Future Regions Research Centre, Federation University of Australia, Berwick, Victoria, Australia

**Keywords:** Antarctica, avian influenza virus, disease ecology, high pathogenicity avian influenza (HPAI), H5N1, penguins, skuas

## Abstract

Despite Antarctica’s geographic isolation, the first incursion of high pathogenicity avian influenza (HPAI) H5N1 was detected in the 2023/2024 austral summer. Surveillance for HPAI H5N1 in Antarctica remains patchy due to logistical, financial and infrastructure challenges, with many suspected cases remaining unconfirmed and few viral genome sequences available to date. Through the 2024/2025 austral summer, we undertook five sampling expeditions to the South Shetland Islands and Antarctic Peninsula facilitated by cruise ships/operators. Across more than 500 faecal environmental samples collected from apparently healthy penguins and marine mammals, we found no detectable evidence of HPAI H5N1. However, HPAI H5N1 was detected in all but one of the skua carcasses sampled, which, in most cases, were found within metres of penguin sub-colonies. All HPAI H5N1 viral genome sequences from skuas on the Antarctic Peninsula fell within a single lineage, which included those genomes from skuas sampled in the 2024/2025 season from the South Shetland Islands. Genomes were in a different clade to those from the Antarctic Peninsula collected in the 2023/2024 austral summer. Our results confirm that although the prevalence may be low, HPAI H5N1 is recurring in Antarctica, emphasizing the need for ongoing surveillance to monitor and mitigate threats to wildlife, even in the planet’s most isolated regions.

## Data Summary

Genomes were deposited in GISAID (EPI_ISL_20303939, EPI_ISL_20303940, EPI_ISL_20303941, EPI_ISL_20303945, EPI_ISL_20303946, EPI_ISL_20303947, EPI_ISL_20304049, EPI_ISL_20304050, EPI_ISL_20304051, EPI_ISL_20304067, EPI_ISL_20304074, EPI_ISL_20304075, EPI_ISL_20304076) and GenBank (accession nos. PZ311845–PZ311948).

**Fig. 1.**
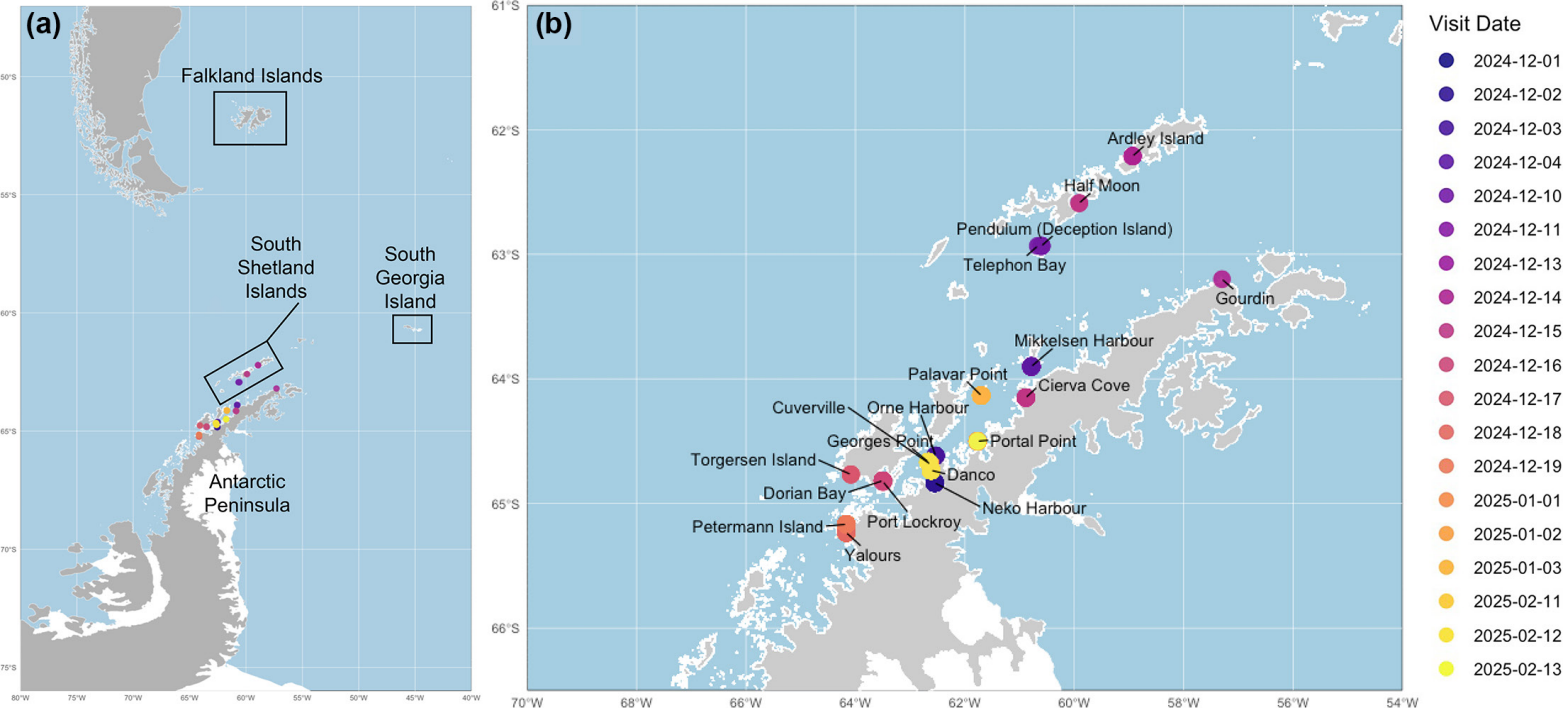
(**a**) Map of key locations, including the Falkland Islands, South Georgia, South Shetland Islands and Antarctic Peninsula. (**b**) Sampling locations in this study. Points are coloured by the sampling date. Details of sampling locations (including latitude and longitude) for each sampling voyage are provided in Table S1.

Phylogenetic tree files and the R script for Fig. 1 are available at https://github.com/michellewille2/Antarctica_24-25/.

**Impact Statement**

High pathogenicity avian influenza (HPAI) H5N1 is causing a global animal pandemic (panzootic), causing catastrophic impacts on all continents but Australia. The virus was first identified in the Antarctic in the austral summer of 2023/2024, a region where surveillance is severely limited by logistical and financial constraints, leaving significant gaps in our understanding of how the virus is spreading across the continent. Herein, we report the results of our surveillance programme through the 2024/2025 austral summer, facilitated by two tour operators. Consistent with previous studies, we detected HPAI H5N1 in found skua carcasses (*Stercorarius* sp.), and we show that brain swabs had a higher viral load compared to oral and cloacal swabs. Phylogenetic analysis demonstrated that the viral genomes we sequenced were similar to those reported from skuas sampled on the South Shetland Islands in the same austral summer but were not in the same clade as those reported from seals on the Antarctic Peninsula. They were also not similar to genomes from the Antarctic Peninsula in the previous austral summer (2023/2024), lending further evidence for multiple viral introductions along the Antarctic Peninsula. As HPAI H5N1 has again been detected in Antarctica in the 2025/2026 season, continued genomic surveillance will be essential for tracking lineage turnover, distinguishing repeated incursions from persistent transmission and ultimately understanding how this virus moves through one of the world’s most ecologically sensitive regions.

## Introduction

High pathogenicity avian influenza (HPAI) H5N1 virus is causing a global panzootic and since 2021 has spread to every continent except Australia [1, 2]. It has been catastrophic for wildlife with detections in over 400 wild bird and 60 wild mammalian species [3, 4]. Beyond a broad host range, HPAI H5N1 has had detrimental population and species-level consequences for some species. For example, 40% of Peruvian Pelicans (*Pelecanus thagus*) in Peru died in 2022, 60% of all Northern Gannets (*Morus bassanus*) across the North Atlantic died in 2022 and 10% of all South American Sea Lions (*Otaria flavescens*) in South America died in 2022–2023 [5–7]. Mass mortality events at this scale have not only species-level impacts, but will certainly also have ecosystem-level impacts, the extent of which will likely only be revealed in the years to come.

Prior to 2023, the remote continent of Antarctica and associated fauna were considered beyond the reach of HPAI H5N1 virus outbreaks. However, Antarctica’s interconnectedness with other regions and particularly South America is increasingly clear, with the emergence of HPAI H5N1 virus in the sub-Antarctic and Antarctica in the 2023/2024 austral summer. HPAI H5N1 virus was first detected in the sub-Antarctic island of South Georgia in October 2023 [8] and was first confirmed on the Antarctic Peninsula in February 2024 [9]. Over the course of the 2023/2024 austral summer, HPAI H5N1 virus was detected across much of the Antarctic Peninsula, with the most southern detection occurring in Marguerite Bay [10]. HPAI H5N1 virus resurged in spring 2024 and was reported along the Antarctic Peninsula throughout the 2024/2025 austral summer [10]. In addition to active circulation in fauna on South Georgia, the Falkland (Malvinas) Islands and the Antarctic Peninsula, the HPAI H5N1 virus spread dramatically through the sub-Antarctic, with detections on the sub-Antarctic Islands of Gough Island, Marion Island, Crozet Island, Kerguelen Island and Heard Island [10–13]. Since its arrival on the Antarctic continent, it has been detected in diverse avian (*n*=11) and mammalian (*n*=5) species in the region. Impacts on the birds and seals of the Falkland Islands and sub-Antarctic islands have been considerable, such as the 47% decline in Southern Elephant Seals (*Mirounga leonina*) on South Georgia [14], mortality of 10,000 Black-browed Albatross (*Thalassarche melanophris*) chicks and adults on the Falkland Islands [15] and the mortality of ~50 adult Snowy (Wandering) Albatross (*Diomedea exulans*) and higher than expected nest failure on South Georgia [16]. Impacts on the charismatic wildlife of the Antarctic continent have been considerably less than anticipated [17] with no mass mortality events in penguins [15]; however, mass mortality events in skuas have occurred [18], skua carcasses positive for HPAI H5N1 have been found as far south as Marguerite Bay (68S) [19] and dead seals have retrospectively tested positive [20], suggesting unclear impacts on marine mammals.

Surveillance for HPAI H5N1 virus in Antarctica poses a number of unique challenges, mostly attributed to the lack of response teams, limited opportunities for repeated visits and the lack of molecular laboratory facilities [21]. Therefore, surveillance in the region is patchy, with both confirmed and suspected cases reported by scientists, citizen scientists and tourist operators [10]. Herein, we report the outcomes of 4 months of surveillance activities undertaken for HPAI H5N1 during the 2024/2025 austral summer in the South Shetland Islands and Antarctic Peninsula aboard two tourist vessels: the MS Ocean Endeavour and MV Argus. This comprises the most comprehensive monitoring study undertaken in the 2024/2025 season. We aimed to reveal whether the HPAI H5N1 virus was present in colonies of apparently healthy penguins, continue to monitor the impacts of this virus on skuas and reveal virus diversity in the region.

## Methods

### Ethics statement

All animal experiments were approved by the Institutional Animal Care and Use Committee at Federation University Australia (AE/2003/004). All research was undertaken in accordance with Australian Antarctic Division permit #ATEP 24/4877.

### Sample collection

Five sample collection expeditions were undertaken between November 2024 and February 2025. Sample collection was facilitated by Intrepid Tours, on the MS Ocean Endeavour vessel, as well as Consulting Antarctica and EVOKN Expedition, on the M/V Argus vessel (Table S1, available in the online Supplementary Material). Samples were collected at 20 sites located on the South Shetland Islands and the Antarctic Peninsula, ranging from the Trinity Peninsula through the Gerlache Strait (Fig. 1).

Non-invasive faecal environmental samples were collected at penguin colonies. To ensure faecal swabs were fresh, penguins were observed defecating and a swab (Copan FloqSwab) of the faeces was collected. Where possible, swabs of fresh pinniped faeces were collected from zodiac boat landing sites and ice floes. From any carcasses found, we collected oropharyngeal, cloacal and/or brain swabs where possible. Swabs were placed into 1 ml of DNA/RNA Shield (Zymo Research) and stored at room temperature on the cruise ship (in alignment with manufacturers’ recommendations). Samples were placed in −80 °C upon arrival in the laboratory (generally within 2 weeks to 1 month of collection).

### Sample screening

RNA was extracted from samples using the QIAGEN QIAmp 96 Virus QIAcube HT Kit (QIAGEN) on a QIAextrator robot. We confirmed that the chemistry of this kit was compatible with DNA/RNA Shield through a comparison of isolates spiked into both virus transport media and DNA/RNA Shield (Zymo Research), with no significant difference in Ct values found. In cases where samples had a substantial amount of faecal material preventing the vacuum from removing all the liquid from the columns during the extraction, they were re-extracted using the Quick-RNA MagBead Kit (Zymo Research) on a KingFisher Apex Instrument (Thermo Fisher). All positive samples were also re-extracted using the Quick-RNA MagBead Kit (Zymo Research).

Extracted RNA was assayed for a short fragment of the conserved matrix gene [22] of influenza A virus using the SensiFAST Probe Lo-Rox qPCR Kit (Bioline Meridian Science) as previously reported [23]. Positive samples were subsequently tested using the CDC A/H5 (Asian Lineage) subtyping Panel (VER 4) and SensiFAST Probe Lo-Rox qPCR Kit (Bioline Meridian Science), with Ct value cut-offs as per the recommended assay guidelines. Positive samples were also tested using a 2.3.4.4b pathotyping assay [24] with the QIAGEN One Step RT-PCR kit and using mastermix and thermocycling conditions described in [24]. Both low pathogenicity avian influenza [A/Turnstone/King Island/7164 CP/2014(H6N8)] and HPAI H5N1 [A/Black-faced Spoonbill/Hong Kong/AFCD-HKU-22-21944-01009/2022(H5N1)] RNA were used as controls for all assays.

### Hybridization capture sequencing

Initial amplicon sequencing (undertaken as per [25]) was not successful, such that we leveraged a hybridization capture sequencing approach. Samples with a Ct value of less than 31 were selected for sequencing, with one sample per carcass attempted (the sample type with the lowest Ct value). Of these, we included ARG071 and ARG072 (reported as A/Brown skua/Torgersen Island/o8182/2024) as positive sequencing controls; they were sequenced in McCulley *et al.* [26] on board the MV Argus within 24 h of collection [26]. These two HPAI H5N1 virus genomes have not been included in subsequent analysis, as they were used as control samples and have been previously reported.

Hybridization capture sequencing was performed using a custom panel (Twist Bioscience, TE-92404697, Table S2), with the Twist Total Nucleic Acids Library Preparation EF Kit 2.0 (Twist Bioscience) for Viral Pathogen Detection and Characterization. The custom probe panel targets seasonal and zoonotic influenza, respiratory syncytial viruses and human metapneumovirus (Table S2 for the list of influenza viruses used in the probe panel design). The hybridization capture sequencing was based on Twist Target Enrichment Standard Hybridization v1 Protocol with modifications listed here: all RNA samples were treated with ezDNase (Invitrogen, Thermo Fisher Scientific) to reduce host genome DNA contamination before double-stranded cDNA synthesis; a reduced volume of one quarter of reagents was used for double-stranded cDNA synthesis, metagenomic library preparation and target enrichment; DNA fragmentation was done at 37 °C for 10 min; Twist UMI Adapters for adapter ligation were used for library preparation. Eight metagenomic libraries were multiplexed into one hybridization capture reaction, and the resulting enriched libraries were sequenced on an Illumina iSeq with 150 bp paired-end reads. Demultiplexing and quality control of reads and genome assembly were undertaken using the IRMA (Iterative Refinement Meta-Assembler) pipeline [27].

### Phylogenetic analysis

All HPAI H5N1 virus genomes from Antarctica, the sub-Antarctic region and the Falkland/Malvinas Islands, as well as the top blast hits for all the genomes generated in this study, were downloaded from Global Initiative on Sharing All Influenza Data (GISAID) (https://gisaid.org/) (11 September 2025). The coding regions of all segments were ordered by descending length (from segment 1 to segment 8) and concatenated. While all genomes included contained all eight segments, some segments were only partial (e.g. genomes from Kerguelen had incomplete PB1 sequences). Sequences were aligned using MAFFT [28] and the E-ins-L algorithm to account for the gaps generated by the incomplete segments, integrated within Geneious Prime (Dogmatics). Maximum-likelihood phylogenetic trees incorporating the best-fit model of nucleotide substitution were estimated using IQ-TREE [29] with 1,000 ultrafast bootstraps on the University of Melbourne (Australia) high-performance computing (HPC) server, Spartan.

The alignment was subsequently filtered to include only genomes for which all segments were complete. We further filtered out genomes comprising different sample types from the same individuals, preferentially retaining either brain or oropharyngeal samples. We evaluated the extent of molecular clock-like structure in the data by performing linear regressions of root-to-tip distances against year of sampling using maximum-likelihood trees using TempEst [30]. As evidence for a molecular clock was obtained, time-scaled phylogenetic trees were estimated using BEAST v1.10.4 [31], under the uncorrelated lognormal relaxed clock [32] and an HKY+G model to accommodate overlapping reading frames in the concatenated genomes. The Bayesian skyline coalescent tree prior was used as this likely reflects the complex epidemiology dynamics of avian influenza viruses through time [33]. The trees were run for 100 million generations on the University of Melbourne HPC, Spartan. Convergence was assessed using Tracer v1.8 (http://tree.bio.ed.ac.uk/software/tracer/). Maximum credibility lineage trees were generated using TreeAnnotator following the removal of 10% burn-in, and trees were visualized using Fig Tree v1.4 (http://tree.bio.ed.ac.uk/software/figtree/).

### Mammalian adaptation

To understand whether there was any evidence of mammalian adaptation in the HPAI H5N1 virus genomes, we scanned genomes for mutations that emerged in South American pinnipeds, listed in [34]. We further interrogated lists of significant mutations generated by FluServer (http://flusurver.bii.a-star.edu.sg), using the ‘automatic detection of closest reference (among current vaccine strains, full genomes)’ option, as well as using A/Baikal teal/KoreaDonglim/3/2014(H5N8) as the reference.

### Analysis and plotting

All analyses were undertaken using R 4.3.3.3 integrated into RStudio 2025.05.1+513 (2025.05.1+513). Maps were made using ggplot2, with the base map from https://data.bas.ac.uk/items/4ecd795d-e038-412f-b430-251b33fc880e/. Ct values were compared using linear mixed models with carcass ID as a random effect using the lme4 package [35]. We restricted the analysis to carcasses from which at least two different sample types were collected.

## Results

### Faecal sampling of apparently healthy animals

Faecal samples (*n*=523) were collected between November 2024 and February 2025 across five expeditions (Tables 1 and S1). Most samples were collected from the Antarctic Peninsula (*n*=507), and in particular, the Gerlache Strait. A further 16 samples were collected from the South Shetland Islands (Fig. 1). Most faecal samples were collected from penguin species, including Adélie Penguin (*Pygoscelis adeliae*; *n*=76), Chinstrap Penguin (*Pygoscelis antarcticus*; *n*=31) and Gentoo Penguin (*Pygoscelis papua*; *n*=340), as well as other avian species in small numbers (Table 1). Faecal environmental samples were also collected from a small number of pinnipeds, including Weddell Seals (*Leptonychotes weddellii*; *n*=15), Antarctic Fur Seals (*Arctocephalus gazella*; *n*=3), Southern Elephant Seals (*Mirounga leonina*; *n*=1) and unidentified seal species (*n*=2). All samples were negative for influenza A virus.

**Table 1.** Faecal environmental samples from apparently healthy animals collected in this study

Date	Location*	Species	# Faecal environmental samples†
1 December 2024	Petermann Island, Antarctic Peninsula	Adélie Penguin *Pygoscelis adeliae*	1
		Gentoo Penguin *Pygoscelis papua*	30
		Skua spp. *Stercorarius* spp.	3
		Weddell Seal *Leptonychotes weddellii*	1
2 December 2024	Cuverville Island, Antarctic Peninsula	Gentoo Penguin *Pygoscelis papua*	27
2 December 2024	Neko Harbour, Antarctic Peninsula	Gentoo Penguin *Pygoscelis papua*	30
		Kelp Gull *Larus dominicanus*	1
3 December 2024	Orne Harbour, Antarctic Peninsula	Gentoo Penguin *Pygoscelis papua*	8
4 December 2024	Mikkelsen Harbour, Antarctic Peninsula	Gentoo Penguin *Pygoscelis papua*	29
		Snowy Sheathbill *Chionis albus*	1
		Southern Elephant Seal (*Mirounga leonina*)	1
		Weddell Seal *Leptonychotes weddellii*	1
10 December 2024	Pendulum, Deception Island, South Shetland Islands	Skua spp. *Stercorarius* spp.	4
		South Polar Skua *Stercorarius maccormicki*	1
10 December 2024	Telefon Bay, Deception Island, South Shetland Islands	Weddell Seal *Leptonychotes weddellii*	1
11 December 2024	Cuverville Island, Antarctic Peninsula	Weddell Seal *Leptonychotes weddellii*	1
13 December 2024	Mikkelsen Harbour, Antarctic Peninsula	Kelp Gull *Larus dominicanus*	1
		Snowy Sheathbill *Chionis albus*	6
14 December 2024	Ardley Island, King George Island, South Shetland Islands	Gentoo Penguin *Pygoscelis papua*	7
14 December 2024	Gourdin, Antarctic Peninsula	Snowy Sheathbill *Chionis albus*	*
15 December 2024	Cierva Cove, Antarctic Peninsula	Gentoo Penguin *Pygoscelis papua*	29
15 December 2024	Half Moon Island, South Shetland Islands	Weddell Seal *Leptonychotes weddellii*	2
15 December 2024	Yankee Harbour, Greenwich Island, South Shetland Islands	Brown Skua *Stercorarius antarcticus*	1
16 December 2024	Dorian Bay, Antarctic Peninsula	Gentoo Penguin *Pygoscelis papua*	30
18 December 2024	Yalour Islands, Antarctic Peninsula	Adélie Penguin *Pygoscelis adeliae*	16
19 December 2024	Petermann Island, Antarctic Peninsula	Gentoo Penguin *Pygoscelis papua*	29
1 January 2025	Danco Island, Antarctic Peninsula	Gentoo Penguin *Pygoscelis papua*	30
2 January 2025	Portal Point, Antarctic Peninsula	Kelp Gull *Larus dominicanus*	29
		Weddell Seal *Leptonychotes weddellii*	6
3 January 2025	Palaver Point, Antarctic Peninsula	Antarctic Shag *Leucocarbo bransfieldensis*	6
		Chinstrap Penguin *Pygoscelis antarcticus*	30
		Weddell Seal *Leptonychotes weddellii*	2
11 February 2025	Petermann Island, Antarctic Peninsula	Adélie Penguin *Pygoscelis adeliae*	30
		Gentoo Penguin *Pygoscelis papua*	34
11 February 2025	Yalour Islands, Antarctic Peninsula	Adélie Penguin *Pygoscelis adeliae*	29
12 February 2025	Danco Island, Antarctic Peninsula	Gentoo Penguin *Pygoscelis papua*	31
		Weddell Seal *Leptonychotes weddellii*	1
12 February 2025	Georges Point, Antarctic Peninsula	Chinstrap Penguin *Pygoscelis antarcticus*	1
		Antarctic Fur Seal *Arctocephalus gazella*	3
		Gentoo Penguin *Pygoscelis papua*	26
		Seal spp.	1
13 February 2025	Portal Point, Antarctic Peninsula	Seal spp.	1

* Locations are presented in Fig. 1, and coordinates are provided in Table S1.

† All samples were negative for HPAI H5N1 virus.

### Overrepresentation of HPAI H5N1 detections in skua carcasses

**Fig. 2.**
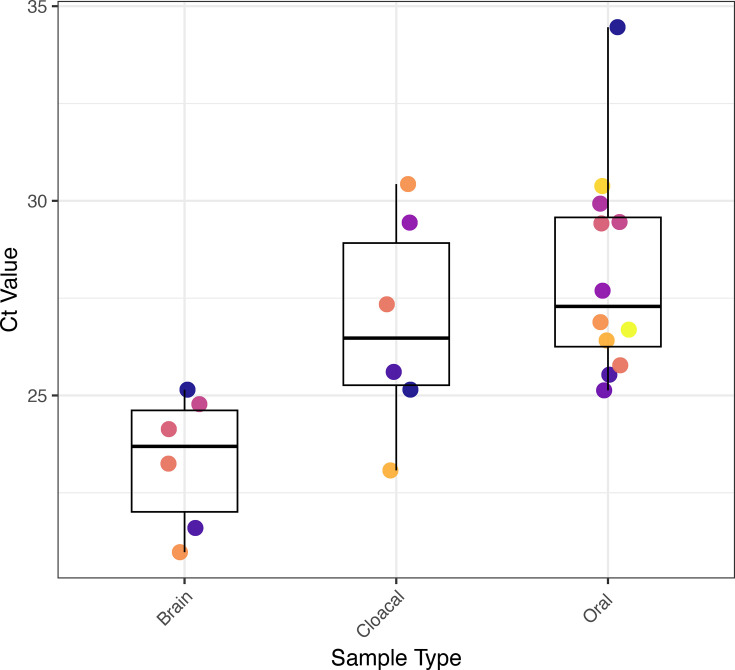
Ct values of different sample types from skua carcasses positive for HPAI H5N1 virus. Different colours correspond to different carcasses tested in this study. Only carcasses from which at least two swabs (from different sample types) have been collected are included. Each carcass comprises a unique colour.

Thirty-four samples (including oropharyngeal, cloacal and/or brain swabs) were collected from 15 carcasses, of which 14 were from skua species (*Stercorarius* spp.) (Table 2). HPAI H5N1 virus was confirmed using a combination of qPCR and, where possible, sequencing in all samples that were positive for influenza A virus. All faecal samples collected from apparently healthy skuas (*n*=10) were negative. For many carcasses, multiple sample types were collected, including brain, oropharyngeal and/or cloacal. Twelve skua carcasses were positive in at least one sample type; two skua carcasses were negative in all sample types. Sample type had a significant effect on Ct values (F₂,₁₇=6.72, *P*<0.05), with cloacal and oropharyngeal swabs having higher Ct values compared to the brain swabs (Fig. 2). In addition to skua carcasses, we also collected brain, oral and anal samples from a single Weddell Seal carcass, sampled at Port Lockroy on 16 December 2024, which was negative for influenza A virus in all sample types.

**Table 2.** Faecal and carcass samples collected from skuas in the 2024/2025 austral summer

Species	Date	Location	Sample source	If carcass, samples collected?	Result*
Skua spp.	1 December 2024	Petermann Island	Faeces		Negative
Skua spp.	1 December 2024	Petermann Island	Faeces		Negative
Skua spp.	1 December 2024	Petermann Island	Faeces		Negative
Skua spp.	2 December 2024	Cuverville Island	Carcass	Oral swab, cloacal swab	Positive
Skua spp.	2 December 2024	Cuverville Island	Carcass	Oral swab, cloacal swab	Negative
Skua spp.	2 December 2024	Cuverville Island	Faeces		Negative
Skua spp.	10 December 2024	Deception Island	Faeces		Negative
Skua spp.	10 December 2024	Deception Island	Faeces		Negative
Skua spp.	10 December 2024	Deception Island	Faeces		Negative
Skua spp.	10 December 2024	Deception Island	Faeces		Negative
South Polar Skua	10 December 2024	Deception Island	Faeces		Negative
Skua spp.	11 December 2024	Cuverville Island	Carcass	Oral swab	Positive
Skua spp.	11 December 2024	Cuverville Island	Carcass	Oral swab, cloacal swab	Positive
Skua spp.	11 December 2024	Cuverville Island	Carcass	Oral swab	Positive
Skua spp.	11 December 2024	Cuverville Island	Carcass	Oral swab	Positive
Brown Skua	15 December 2024	Yankee Harbour	Faeces		Negative
South Polar Skua	17 December 2024	Torgersen Island	Carcass††	Oral swab, cloacal swab brain swab, lung swab	Positive
Skua spp.	2 January 2025	Cuverville Island	Carcass	Oral swab, cloacal swab brain swab	Positive
Skua spp.	2 January 2025	Cuverville Island	Carcass	Oral swab, brain swab	Positive
Skua spp.	2 January 2025	Cuverville Island	Carcass	Oral swab, brain swab	Positive
Skua spp.	2 January 2025	Cuverville Island	Carcass	Oral swab, cloacal swab brain swab,	Positive
Skua spp.	2 January 2025	Cuverville Island	Carcass	Oral swab, cloacal swab brain swab	Positive
Skua spp.	11 February 2025	Yalour Islands	Carcass	Oral swab, brain swab, tissue swab	Positive
Skua spp.	12 February 2025	Danco Island	Carcass	Oral swab	Negative

* Carcasses were deemed positive if any one sample tested positive.

† Carcass reported in [26].

One Brown skua (*Stercorarius antarcticus*) carcass sampled on the Yalour Islands was banded, and a query of the banding record indicated the bird was originally banded at Palmer Station in 2001, as a chick, by researchers part of the US Antarctic Program. Thus, this individual was 23 years old and was positive for HPAI H5N1. As no other birds were banded, it is unclear whether young or adult birds are being disproportionately impacted.

### Viral genomes from 2024/2025 are not in the same clade as those from the previous season

We attempted to sequence at least one sample from each positive carcass, with samples selected having a Ct range of 23–31. Initial attempts with amplicon sequencing using standard approaches were not successful; however, we successfully generated full genome sequences from 15 samples using a custom hybrid capture panel approach. Of the 16 positive samples we attempted to sequence, only 1 failed (read depth below depth threshold, INT-OE5-452, matrix Ct=29). For successfully sequenced samples, read depth ranged from a median of 166 (INT-OE5-275, PB1) to 37795 (INT-OE5-281 HA) (Table S3). Both positive control samples (ARG071 and ARG072, which were sequenced in McCulley *et al*. [26]) were successfully sequenced, validating our use of this sequencing approach. We do not discuss these two HPAI H5N1 genomes further, given they have already been reported.

The genotype of all viruses generated in this study was consistent with B3.2 (as per the GenoFLU nomenclature, https://github.com/USDA-VS/GenoFLU), which are descended from the main introduction of B3.2 from North America into South America and subsequently Antarctica [36]. Further, within the South American clade, the viruses sequenced here fall into the clade of Antarctic sequences, which is descended from the ‘Avian Clade’ of the B3.2 South American introduction (i.e. clade I from [37]), rather than the ‘marine mammal-adapted clade’ (i.e. the clade containing a PB2 D701N mutation, as defined by [34]).

**Fig. 3.**
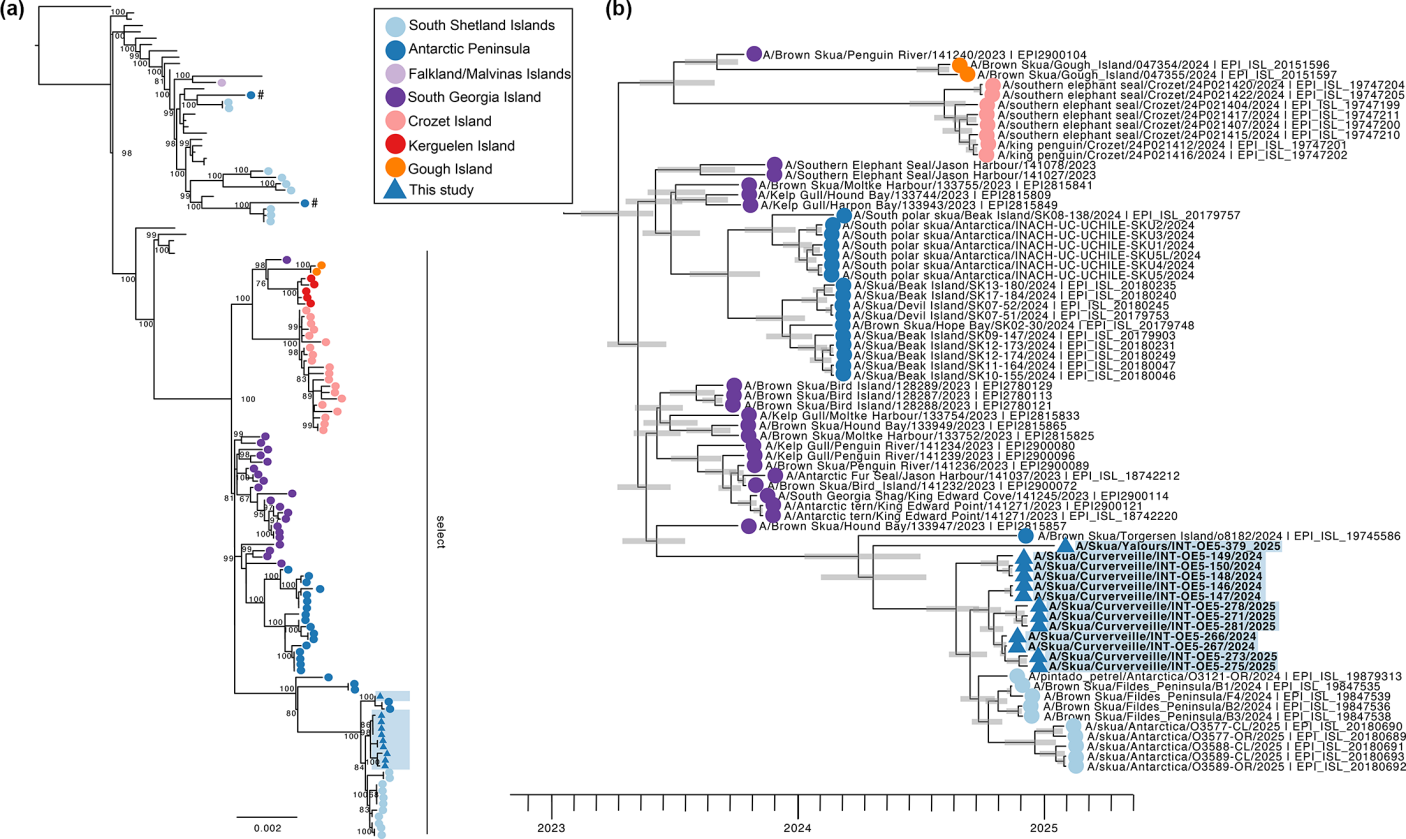
Phylogenetic tree of HPAI H5N1 virus from the Antarctic Peninsula. (**a**) Maximum-likelihood tree of the concatenated genomes of HPAI H5N1 in the sub-Antarctic and Antarctica. Bootstrap values are presented at major branches. Scale bar indicates the number of substitutions per site. A/red fox/Michigan (same GenoFlu genotype, B3.2) is used as the outgroup. (**b**) Time-structured tree of the concatenated coding complete genomes of the sub-Antarctic/Antarctic clade. Time scale is in years. Node bars comprise 95% highest posterior density of node height. Filled circles comprise the location. Genomes from this study are indicated by blue triangles, and tip names are within blue boxes. (**a, b**) Genomes from GISAID downloaded as of 30 November 2025 and for (**a**) supplemented by [20], denoted by a hash (#).

The concatenated HPAI H5N1 viral genomes from skuas sampled in Cuverville Island (*n*=12) shared 99.9% pairwise identity, and these genomes clustered together in phylogenetic trees (Fig. 3a). Time-structured phylogeny (Fig. 3b) suggests these genomes fall into two small sub-clades, where INT-OE5-148, 149 and 150 are sister to a clade containing all the other genomes from Cuverville Island, as well as sequences from the South Shetland Islands. Carcasses from Cuverville Island were sampled from December 2024 to February 2025, and sequences from 2024 and 2025 do fall into these different sub-clades. The genome from a skua sampled on the Yalour Islands in February 2025 did not fall into this cluster; rather, it was sister to the clade containing genomes from Cuverville Island and the South Shetlands (Fig. 3). However, the concatenated genome was 99.7% to that from Cuverville Island and 99.8% similar to that from Torgersen Island reported in [26].

Two HPAI H5N1 virus partial genomes published by Younger *et al* [20] reported from the 2024/2025 season, from Crab-eater Seals (*Lobodon carcinophaga*) and Kelp Gulls (*Larus dominicanus*), did not fall into the clade comprising genomes generated in this study but rather were related to genomes from the South Shetland Islands, which fell into the broader Antarctic clade (clade II, as defined by [37]), which is descended from the ‘South American marine mammal’ clade (as defined by [34]) (Fig. 3a). This suggests the potential for further undetected diversity in the region.

Overall, the genomes determined in this study are not in the same clade as those previously reported from the Antarctic Peninsula, all of which were from the 2023/2024 season (Fig. 3). This suggests that rather than maintenance of the 2023/2024 lineage on the peninsula, an independent incursion occurred coinciding with the 2024/2025 season, confirming complex patterns of virus movement in the region.

### No evidence for mammalian-adapted mutations, but the NA stalk deletion may affect NA glycosylation

No mutations associated with putative mammal-to-mammal transmission in South American pinnipeds (listed in [34]), or PB2 E627K, were present in any of the genomes generated in this study. Using FluServer, the host specifies shift mutations HA T110S and V226A were identified, but only when using H5N8_Baikalteal_2014_KoreaDonglim as a reference, and not when using H5N1_AmericanWigeon_2021_SouthCarolina22-000345-00 (suggesting these mutations are not unique to these viruses). Mutations associated with changes in virulence (PB2 R339K, PB1 T375S, HA S157P, M K101R, and NS L19M, P167S), antigenic drift (NA T65del and S366I and HA L131Q and S157P) and the loss of glycosylation sites (the 51 nt deletion in NA; NA N58del, N63del, N68del, S70del, T60del, T65del) were identified (Table S3). Many of these mutations in the NA segment were deletions, associated with the 51 bp deletion, which we and others have observed in some genomes from the Antarctic [26, 38, 39] (Fig. S1).

## Discussion

HPAI H5N1 virus is having a catastrophic impact on wildlife globally, with considerable concerns for the unique and charismatic Antarctic wildlife. Indeed, there have been considerable impacts on wildlife in the sub-Antarctic, including the 50% population reduction in the Southern Elephant Seals (*Mirounga leonina*) population in 2023/2024 on South Georgia Island [14]; ~3,000 Black-browed Albatross (*Thalassarche melanophris*) adults found dead at Jason Steeple Island (Falkland Islands) in 2023/2024 [40] and more recently a further ~40 dead Black-browed Albatross on Beauchêne Island (Falkland Islands) [41]. However, comparable impacts have not been observed in Antarctica. It is clear that skuas are an important indicator species as skua mortality has proven to be the most reliable early indicator of HPAI H5N1 virus presence, [e.g. 12], and carcasses have tested positive at almost every site tested throughout the Antarctic Peninsula [10]. In addition to Antarctic skua species, Great Skuas (*Stercorarius skua*) in the northern hemisphere have also been heavily impacted [42, 43]. Indeed, all positives reported in this study are from skua carcasses. No faecal samples from apparently healthy skuas, or any other species, were positive, despite carcasses being found mere metres from penguin colonies. When carcasses were tested for HPAI H5N1 virus, we consistently found that brain samples had the lowest Ct values, which is consistent with neurotropism reported across avian and mammalian species, e.g. [44–47]. Indeed, in histology of HPAI H5N1 virus-positive skuas examined during the 2024 Australis Expedition [44], 5/6 had influenza A virus nucleoprotein antigen present in the brain (via immunohistochemistry). Additionally, 2/5 skuas also had influenza A virus nucleoprotein in the lungs, and one tested positive across a number of other organs [44]. Despite this clear impact on skuas [18, 19, 48–51], quantitative information on skua population size and breeding success remains scarce for most sites [52–58], which prevents evaluation of whether observed mortality has translated into a population-level decline. Resolving this uncertainty is essential for developing informed conservation and management strategies for these sentinel species.

In the >500 samples from apparently healthy penguins across a diversity of colonies, locations and species, we did not detect HPAI H5N1 virus in any of these samples. At present, it is unclear the role that penguins play in HPAI H5N1 virus ecology. In the sub-Antarctic, the HPAI H5N1 virus has caused mortality events in King Penguins (*Aptenodytes patagonicus*) and Gentoo Penguins [10, 11, 51]. In South Africa, HPAI H5N1 has caused mortality in African Penguins (*Spheniscus demersus*) [59], and in Peru, Humboldt Penguins (*Spheniscus humboldti*) [60], confirming that Sphenisciformes are susceptible, and if exposed, incur considerable mortality. Along the Antarctic Peninsula, it appears that penguin species have not observed HPAI H5N1-related mortality events, given no observations of large numbers of dead penguins or observed colony decline across years in monitored colonies [10]. As to whether penguins are infected with HPAI H5N1 without obvious disease remains unclear, as some studies report HPAI H5N1 virus in apparently healthy penguins [61], and others do not [18]. Given the uncertainty around the outcome of molecular testing results, serology will be critical in clarifying the role of penguins. Indeed, a recent study found no detectable H5 antibodies in any of the Gentoo, Chinstrap or Adélie Penguins tested [20]. Clarifying whether penguins have subclinical infections is critical. Infected animals with no clinical disease play an important role in virus spread. For example, Mallards (*Anas platyrhynchos*), which have limited disease signs, have been implicated in the long-distance spread of HPAI H5N1 in the northern hemisphere [62, 63]. If penguin species have subclinical infections, they may play a similar role in virus spread and maintenance and should be targeted for far more surveillance than at present. As evidenced by studies in Mallards, [e.g. 64], serology is a powerful approach in helping to resolve viral dynamics in apparently healthy birds and should be better leveraged in current surveillance. Despite this, serology has largely been absent from published studies from the Antarctic (to date) due to a number of barriers, including the requirement for expanded animal ethics beyond sampling carcasses or faeces, the requirement for specialised training and continued challenges in validating serological methods for wildlife. Further, in most countries, neither active nor passive surveillance has a strong serological component, such that it is a lower priority compared to sample collection for molecular detection.

Given the enormous challenges for HPAI H5N1 virus surveillance on the continent, viral genome sequences are critical in revealing virus circulation and movement dynamics. That the HPAI H5N1 genomes from the 2024/2025 austral summer are not in the same clade as those from 2023/2024 suggests that HPAI H5N1 virus suggest reintroduction to the Antarctic Peninsula, most plausibly from South Georgia Island. Whether HPAI H5N1 is persisting in the environment between seasons is not known and warrants attention. Limited genomic representation from other regions, including South Georgia Island, prevents firm conclusions about source populations or transmission routes. Overall, this highlights the urgent need for sustained, coordinated genomic surveillance to build a more coherent understanding of HPAI H5N1 virus emergence and movement across the Antarctic region.

## Supplementary material

10.1099/mgen.0.001724 Supplementary Material 1.

10.1099/mgen.0.001724 Supplementary Material 2.

## Abbreviations

Ct: cycle threshold
Global Initiative on Sharing All Influenza Data: GISAID
HPAI: high pathogenicity avian influenza
HPC: high-performance computing

## References

[1] Wille M, Atkinson R, Barr IG, Burgoyne C, Bond AL (2024). Long‐distance avian migrants fail to bring 2.3.4.4b HPAI H5N1 Into Australia for a second year in a row. Influenza Resp Viruses.

[2] Bellido-Martín B, Rijnink WF, Iervolino M, Kuiken T, Richard M (2026). Evolution, spread and impact of highly pathogenic H5 avian influenza A viruses. Nat Rev Microbiol.

[3] Klaassen M, Wille M (2023). The plight and role of wild birds in the current bird flu panzootic. *Nat Ecol Evol*.

[4] Peacock TP, Moncla L, Dudas G, VanInsberghe D, Sukhova K (2025). The global H5N1 influenza panzootic in mammals. Nature.

[5] Matthiopoulos J, Votier S, Wanless S, Cunningham E, Lane J (2025). Multispecies HPAI spillovers led to the death of one-in-three breeding gannets. ResearchSquare.

[6] Kuiken T, Vanstreels RET, Banyard A, Begeman L, Breed AC (2026). Emergence, spread, and impact of high-pathogenicity avian influenza H5 in wild birds and mammals of South America and Antarctica. Conserv Biol.

[7] Leguia M, Garcia-Glaessner A, Muñoz-Saavedra B, Juarez D, Barrera P (2023). Highly pathogenic avian influenza A (H5N1) in marine mammals and seabirds in Peru. Nat Commun.

[8] Banyard AC, Bennison A, Byrne AMP (2024). Detection and spread of high pathogenicity avian influenza virus H5N1 in the Antarctic Region. Nat Commun.

[9] CIENCIA - Ministerio de Ciencia IyU (2024). Científicos del Centro de Biología Molecular Severo Ochoa del CSIC confirman la presencia por primera vez en la Antártida del virus de la Gripe Aviar Altamente Patogénica. Accessed 29/20/2025. https://www.ciencia.gob.es/Noticias/2024/febrero/gripe-aviar-antartida.html.

[10] SCAR Antatctic Wildllife Health Action Group (2025). Sub-Antarctic and Antarctic Highly Pathogenic Avian Influenza H5N1 Monitoring Project. Accessed 29/10/2025. https://scar.org/library-data/avian-flu.

[11] Clessin A, Briand F-X, Tornos J, Lejeune M, De Pasquale C (2025). Circumpolar spread of avian influenza H5N1 to southern Indian Ocean islands. Nat Commun.

[12] Steinfurth A, Lynton-Jenkins JG, Cleeland J, Mollett BC, Coombes HA (2025). Investigating high pathogenicity avian influenza virus incursions to remote islands: detection of H5N1 on Gough Island in the South Atlantic Ocean. bioRxiv.

[13] Watt M (2025). Joint media release: Confirmation of H5 bird flu on sub-Antarctic Heard Island. https://minister.dcceew.gov.au/watt/media-releases/joint-media-release-confirmation-h5-bird-flu-sub-antarctic-heard-island.

[14] Bamford CCG, Fenney N, Coleman J, Fox-Clarke C, Dickens J (2025). Highly Pathogenic Avian Influenza Viruses (HPAIV) Associated with Major Southern Elephant Seal Decline at South Georgia. Commun Biol.

[15] SCAR (2024). Updated biological risk assessment and recommendations for highly pathogenicity avian influenza in Antarctica. Report prepared for Scientific Committee on Antarctic Research. https://scar.org/~documents/route%3A/download/6304.

[16] Bennison A, Adlard S, Banyard AC, Blockley F, Blyth M (2024). A case study of highly pathogenic avian influenza (HPAI) H5N1 at Bird Island, South Georgia: the first documented outbreak in the subantarctic region. Bird Study.

[17] Wille M, Dewar M (2026). The spread of high pathogenicity avian influenza H5N1 to Antarctica. Antarctic Environ Portal.

[18] Aguado B, Begeman L, Günther A, Iervolino M, Soto F (2024). Searching for high pathogenicity avian influenza virus in Antarctica. Nat Microbiol.

[19] Gorta SBZ, Neira V, Wille M, Gajardo D, Yılmaz A (2025). Impacts of high pathogenicity avian influenza H5N1 2.3.4.4b south of the Antarctic circle. bioRxiv.

[20] Younger JL, Patier L, Brav-Cubitt T, van Tonder A, Clessin A (2026). High pathogenicity avian influenza virus H5N1 clade 2.3.4.4b in Antarctica: multiple introductions and the first confirmed infection of ice-dependent seals. bioRxiv.

[21] Wille M, Dewar ML, Claes F, Thielen P, Karlsson EA (2025). A call to innovate Antarctic avian influenza surveillance. Trends Ecol Evol.

[22] Spackman E, Senne DA, Myers TJ, Bulaga LL, Garber LP (2002). Development of a real-time reverse transcriptase PCR assay for type A influenza virus and the avian H5 and H7 hemagglutinin subtypes. J Clin Microbiol.

[23] Wille M, Lisovski S, Roshier D, Ferenczi M, Hoye BJ (2022). Strong phylogenetic and ecological effects on host competency for avian influenza in Australian wild birds. Proc R Soc B.

[24] James J, Seekings AH, Skinner P, Purchase K, Mahmood S (2022). Rapid and sensitive detection of high pathogenicity Eurasian clade 2.3.4.4b avian influenza viruses in wild birds and poultry. J Virol Methods.

[25] Wille M, Atkinson R, Barr IG, Bond AL, Boyle D (2025). Surveillance of migratory shorebirds and seabirds in 2024 in Australia reveals incursions of a diversity of low pathogenicity avian influenza viruses though not high pathogenicity avian influenza H5N1. bioRxiv.

[26] McCulley M, Dewar ML, Low YS, Wilson A, Jauregui R (2025). High pathogenicity avian influenza (HPAI) H5N1 virus detected in brown Skua using portable laboratory while at sea in Antarctica. Microbiol Resour Announc.

[27] Shepard SS, Meno S, Bahl J, Wilson MM, Barnes J (2016). Viral deep sequencing needs an adaptive approach: IRMA, the iterative refinement meta-assembler. BMC genomics.

[28] Katoh K, Standley DM (2013). MAFFT multiple sequence alignment software version 7: improvements in performance and usability. Mol Biol Evol.

[29] Nguyen L-T, Schmidt HA, von Haeseler A, Minh BQ (2015). IQ-TREE: a fast and effective stochastic algorithm for estimating maximum-likelihood phylogenies. Mol Biol Evol.

[30] Rambaut A, Lam TT, Max Carvalho L, Pybus OG (2016). Exploring the temporal structure of heterochronous sequences using TempEst (formerly Path-O-Gen). Virus Evol.

[31] Drummond AJ, Suchard MA, Xie D, Rambaut A (2012). Bayesian phylogenetics with BEAUti and the BEAST 1.7. Mol Biol Evol.

[32] Li WLS, Drummond AJ (2012). Model averaging and Bayes factor calculation of relaxed molecular clocks in Bayesian phylogenetics. Mol Biol Evol.

[33] Drummond AJ, Rambaut A, Shapiro B, Pybus OG (2005). Bayesian coalescent inference of past population dynamics from molecular sequences. Mol Biol Evol.

[34] Uhart MM, Vanstreels RET, Nelson MI, Olivera V, Campagna J (2024). Epidemiological data of an influenza A/H5N1 outbreak in elephant seals in Argentina indicates mammal-to-mammal transmission. Nat Commun.

[35] Bates D, Mächler M, Bolker B (2015). Fitting linear mixed-effects models using lme4. J Stat Softw.

[36] Signore AV, Giacinti J, Jones MEB, Erdelyan CNG, McLaughlin A (2025). Spatiotemporal reconstruction of the North American A(H5N1) outbreak reveals successive lineage replacements by descendant reassortants. Sci Adv.

[37] Clessin A, Brusselmans M, Hong SL, Tornos J, Lejeune M (2026). Dispersal, adaptation and persistence of H5N1 in the sub-Antarctic and Antarctica. *bioRxiv*.

[38] Ogrzewalska M, Vanstreels RT, Pereira E, Campinas E, Junior LC (2025). Genomic analysis of high pathogenicity avian influenza viruses from Antarctica reveals multiple introductions from South America. ResearchSquare.

[39] Ruifeng X, Minhao G, Nailou Z, Zhenhua W, Zheng W (2025). Transcontinental spread of HPAI H5N1 from South America to Antarctica via avian vectors. bioRxiv.

[40] Kuepfer A, Stanworth A Falkland Islands seabird monitoring programme annual report 2023/2024 2024:73 pages. https://falklandsconservation.com/wp-content/uploads/2024/10/Kuepfer-Stanworth-2024-FISMP-2023_24.pdf.

[41] ACAP (2025). Highly Pathogenic Avian Influenza confirmed among Black-browed Albatrosses on Beauchêne Island in the South Atlantic. https://www.acap.aq/latest-news/the-presence-of-highly-pathogenic-avian-influenza-has-been-confirmed-on-beauchene-island-in-the-south-atlantic-home-of-the-worlds-second-largest-black-browed-albatross-colony.

[42] Banyard AC, Lean FZX, Robinson C, Howie F, Tyler G (2022). Detection of highly pathogenic avian influenza virus H5N1 Clade 2.3.4.4b in Great Skuas: a species of conservation concern in Great Britain. Viruses.

[43] Camphuysen KCJ, Furness RW (2022). Avian influenza leads to mass mortality of adult great skuas in foula in summer 2022. Scotish Birds.

[44] Iervolino M, Günther A, Begeman L, Aguado B, Bestebroer TM (2025). The expanding avian influenza panzootic: skua die-off in Antarctica. bioRxiv.

[45] Bordes L, Germeraad EA, Roose M, van Eijk NMHA, Engelsma M (2024). Experimental infection of chickens, Pekin ducks, Eurasian wigeons and Barnacle geese with two recent highly pathogenic avian influenza H5N1 clade 2.3.4.4b viruses. Emerg Microb Infect.

[46] Caliendo V, Leijten L, van de Bildt M, Germeraad E, Fouchier RAM (2022). Tropism of highly pathogenic avian influenza H5 Viruses from the 2020/2021 epizootic in wild ducks and geese. Viruses.

[47] Larsen SV, Israelson R, Torp C, Larsen LE, Jensen HE (2025). Transmission, pathological and clinical manifestations of highly pathogenic avian influenza A virus in mammals with emphasis on H5N1 Clade 2.3.4.4b. Viruses.

[48] León F, Ulloa-Contreras C, Pizarro EJ, Castillo-Torres PN, Díaz-Morales KB (2025). Skuas mortalities linked to positives HPAIV A/H5 beyond polar Antarctic circle. bioRxiv.

[49] Neira V, Ariyama N, Castillo-Torres PN, Brito B, Muñoz G (2025). Genetic Characterization of highly pathogenic avian influenza A(H5N1) clade 2.3.4.4b, Antarctica, 2024. Emerg Infect Dis.

[50] Bennett-Laso B, Berazay B, Muñoz G, Ariyama N, Enciso N (2024). Confirmation of highly pathogenic avian influenza H5N1 in skuas, Antarctica 2024. Front Vet Sci.

[51] Dewar M, Alcami A, Wille M (2024). Updated biological risk assessment and recommendations for highly pathogenicity avian influenza in Antarctica. SCAR Bulletin. https://scarorg/~documents/route%3A/download/6304.

[52] Ainley DG, Morrell SH, Wood RC (1986). South Polar skua breeding colonies in the Ross Sea region, Antarctica. *Notornis*.

[53] Komarowska K, Fudala K, Dziembowski M, Hagge A, Bialik RJ (2025). Population status of sympatrically breeding skuas (*Catharacta* spp.) at Admiralty Bay, King George Island, Antarctica: a Case Report for 2020–2024. Biology.

[54] Pascoe JG (1984). A census of the South Polar skua at Cape Hallett, Antarctica. Notornis.

[55] Santa Cruz F, Krüger L (2023). Breeding population and nesting habitat of skuas in the harmony point antarctic specially protected area. Diversity.

[56] Wilson DJ, Lyver PO, Greene TC, Whitehead AL, Dugger KM (2017). South polar skua breeding populations in the Ross Sea assessed from demonstrated relationship with Adélie Penguin numbers. Polar Biol.

[57] Wilson KJ, Turney C, Fogwill C (2015). Low numbers and apparent long-term stability of south polar skuas *Stercorarius maccormicki* at commonwealth bay, Antarctica. Marine Ornithol.

[58] Wood RC (1971). Population dynamics of breeding south polar skuas of unknown age. The Auk.

[59] Molini U, Aikukutu G, Roux J-P, Kemper J, Ntahonshikira C (2020). Avian influenza H5N8 outbreak in African Penguins (*Spheniscus demersus*), Namibia, 2019. J Wildl Dis.

[60] Muñoz G, Ulloa M, Alegría R, Quezada B, Bennett B (2024). Stranding and mass mortality in Humboldt Penguins (*Spheniscus humboldti*), associated to HPAIV H5N1 outbreak in Chile. Prev Vet Med.

[61] León F, Le Bohec C, Pizarro EJ, Baille L, Cristofari R (2025). Tracking HPAIV H5 through a geographic survey of Antarctic seabird populations. Sci Rep.

[62] Lv X, Li X, Sun H, Li Y, Peng P (2022). Highly pathogenic avian influenza A(H5N8) clade 2.3.4.4b Viruses in Satellite-Tracked Wild Ducks, Ningxia, China, 2020. Emerg Infect Dis.

[63] Teitelbaum CS, Masto NM, Sullivan JD, Keever AC, Poulson RL (2023). North American wintering mallards infected with highly pathogenic avian influenza show few signs of altered local or migratory movements. Sci Rep.

[64] Wight J, Rahman I, Wallace HL, Cunningham JT, Roul S (2024). Avian influenza virus circulation and immunity in a wild urban duck population prior to and during a highly pathogenic H5N1 outbreak. Vet Res.

