# Supplementary material for: Skuas as sentinels of high pathogenicity avian influenza H5N1 on the Antarctic Peninsula in the 2024/2025 austral summer
Source: Microb Genom. 2026 Aug 14;12(8):001724. doi: 10.1099/mgen.0.001724 (PMC13476044; doi:10.1099/mgen.0.001724)
Supplement: Supplementary file 1 [file mgen-12-01724-s001.pdf]

Skuas as sentinels of high pathogenicity avian influenza H5N1 on the Antarctic Peninsula in the 2024/2025 austral summer

Michelle Wille, Will Abbott, Darrel Day, Yi-Mo Deng, Xiaomin Dong, Ted Gibson, Ryan Hope-Inglis, Michelle McCulley, Ida Olsson, Arvind Varsani, Teri Visintin, Matthew Walters, Meagan Dewar

SUPPLEMENTAL FIGURES AND TABLES

Supplemental Table/Figure	Page
**Table S1**. Sampling expeditions.	1
**Table S2**. Influenza A genomes used for initial design of TWIST panel	2
**Figure S1**. Coverage map for a representative genome generated in this study and NA alignment.	3
**Table S3**. Ct values, coverage statistics, and accession numbers for positive samples from Skuas	See separate excel sheet
**Table S4.** Mutations of note identified using Flu Server	See separate excel sheet

Table S1. Sampling expeditions.

Expedition	Date	Location	Location	Latitude	Longitude	# samples collected
OE5-03	1/12/2024	Antarctic Peninsula	Petermann Island	-65.16667	-64.16667	35
OE5-03	2/12/2024	Antarctic Peninsula	Cuverville Island	-64.68333	-62.63333	32
OE5-03	2/12/2024	Antarctic Peninsula	Neko Harbour	-64.83333	-62.55	30
OE5-03	2/12/2024	South Shetland Islands	Neko Harbour	-64.83333	-62.55	1
OE5-03	3/12/2024	Antarctic Peninsula	Orne Harbour	-64.61667	-62.53333	8
OE5-03	4/12/2024	Antarctic Peninsula	D'Hainaut Island (Mikkelsen Harbour)	-63.9	-60.78333	33
OE5-04	10/12/2024	South Shetland Islands	Pendulum (Deception Island)	-62.9333	-60.6	5
OE5-04	10/12/2024	South Shetland Islands	Telefon Bay	-62.93333	-60.66667	1
OE5-04	11/12/2024	Antarctic Peninsula	Cuverville Island	-64.68333	-62.63333	6
OE5-04	13/12/2024	Antarctic Peninsula	D'Hainaut Island (Mikkelsen Harbour)	-63.9	-60.78333	7
OE5-04	14/12/2024	Tip of Peninsula	Gourdin	-63.2	-57.3	1
OE5-04	15/12/2024	South Shetland Islands	Half Moon	-62.59	-59.91	2
OE5-04	15/12/2024	South Shetland Islands	Yankee Harbour	-59.78333	-62.53333	1
Argus	14/12/2024	South Shetland Islands	Ardley Island	-62.21308	-58.93317	7
Argus	15/12/2024	Antarctic Peninsula	Cierva Cove	-64.15	-60.88333	29
Argus	16/12/2024	Antarctic Peninsula	Dorian Bay	-64.81667	-63.5	30
Argus	16/12/2024	Antarctic Peninsula	Port Lockroy	-64.82528	-63.49444	3
Argus	17/12/2024	Antarctic Peninsula	Torgersen Island	-64.76667	-64.08333	4
Argus	18/12/2024	Antarctic Peninsula	Yalour Islands	-65.23333	-64.16667	16
Argus	19/12/2024	Antarctic Peninsula	Petermann Island	-65.16667	-64.16667	29
OE5-06	1/1/2025	Antarctic Peninsula	Danco Island	-64.73333	-62.61667	30
OE5-06	2/1/2025	Antarctic Peninsula	Cuverville Island	-64.68333	-62.63333	13
OE5-06	2/1/2025	Antarctic Peninsula	Portal Point	-64.5	-61.76667	36
OE5-06	3/1/2025	Antarctic Peninsula	Palavar Point (Two Hummock Island)	-64.13333	-61.7	38
OE5-07	11/2/2025	Antarctic Peninsula	Petermann Island	-65.16667	-64.16667	64
OE5-07	11/2/2025	Antarctic Peninsula	Yalour Islands	-65.23333	-64.16667	33
OE5-07	12/2/2025	Antarctic Peninsula	Danco Island	-64.73333	-62.61667	33
OE5-07	12/2/2025	Antarctic Peninsula	Georges Point	-64.66667	-62.66667	31
OE5-07	13/2/2025	Antarctic Peninsula	Portal Point	-64.5	-61.76667	1

Table S2. Influenza A genomes used for initial design of TWIST panel. The influenza B virus, respiratory syncytial viruses (RSV) and human metapneumovirus (hMPV) genomes incorporated are not shown.

Subtype	Clade	GISAID Acc No.(EPI_ISL_)	Designation
**H5N1**	2.3.2.1a	19156871	A/Victoria/149/2024
	2.3.2.1c	18373263	A/Cambodia/NPH230776/2023
	2.3.2.1b	96862	A/duck/Zhejiang/2244/2011
	2.3.4.4b	19723001	A/red-fox/Italy/25VIR819-1/2024
	2.3.4.4b	19027114	A/Texas/37/2024
	LPAI	18800494	A/Chicken/Netherlands/24000703-016020/2024
**H7**		19231583	A/Chicken/Victoria/10-17/2024
		18593866	A/Chicken/Netherlands/23069-021/2023
		285291	A/Sichuan/27180/2017
		14769181	A/Wild_duck/Victoria/15-0591-15/2015
	Eurasian lineage HPAI	19519487	A/chicken/Germany-NI/2024AI02733/2024
		332358	A/chicken/Jiangsu/1/2018
		295523	A/duck/Cambodia/b0120501/2017
	North American lineage HPAI	16209261	A/chicken/Guanajuato/CPA-01819-22/2022
**H9**	G5.5	406471	A/Senegal/0243/2019
	G5.7	140388	A/Bangladesh/0994/2011
	B4.7	18377814	A/Anhui-Tianjiaan/11086/2022
	B4.7	17068774	A/Cambodia/AHC220057/2022
	B4.6	18900599	A/chicken/South_Korea/2330076/2023
**H1N1pdm09**	5a.2a	19812950	A/Victoria/121/2025
	5a.2a.1	19465945	A/Victoria/4897/2022
		19030767	A/Sydney/1/2024
		19440424	A/Wisconsin/588/2019
**H3N2**	2a.3a.1	19561250	A/Canberra/614/2024
	2a.3a.1	19558653	A/Massachusetts/18/2022
		2233240	A/Darwin/9/2021

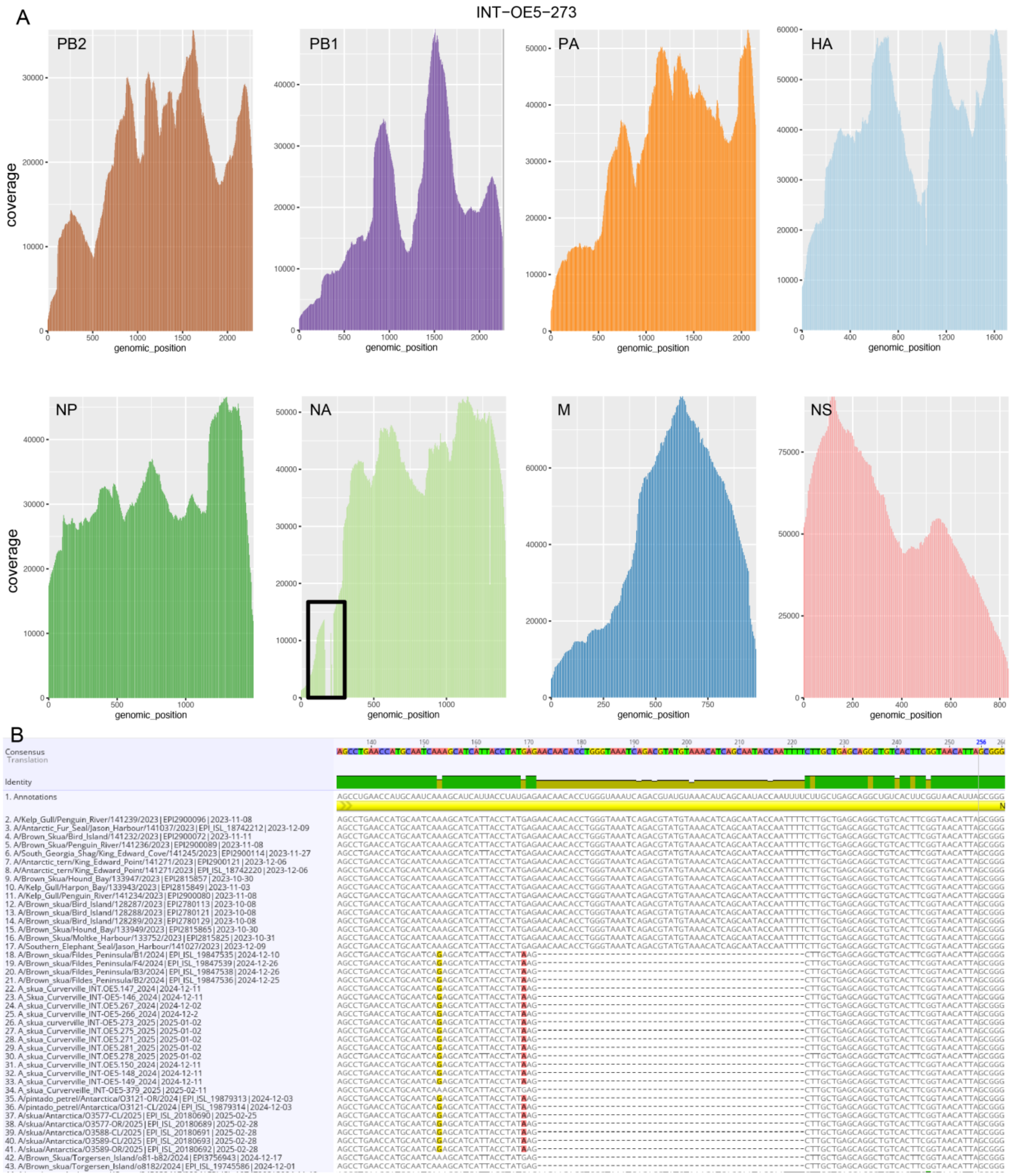

Figure S1. (A) Coverage map for a representative genome generated in this study, A/Skua/Curverville/INT-OE5-273/2024(H5N1). Each panel comprises a segment. For the NA segment, across all genomes, a 51 bp detection was detected relative to the reference genomes. Mean coverage for all genomes is provided in Table S3 (B) Excerpt of alignment of the NA-N1 of sequences from the sub-Antarctic and Antarctica demonstrating this deletion to occur in genomes from [26, 37, 38], suggesting a *bona fide* deletion.

Table S3. Ct values, coverage statistics, and accession numbers for positive samples from Skuas.

See attached excel spreadsheet

Table S4. Mutations of note identified using Flu Server.

See attached excel spreadsheet
